# Small Scale, High Precision: Robotic Surgery in Neonatal and Pediatric Patients—A Narrative Review

**DOI:** 10.3390/children11030270

**Published:** 2024-02-21

**Authors:** Emil Radu Iacob, Roxana Iacob, Laura Andreea Ghenciu, Tudor-Alexandru Popoiu, Emil Robert Stoicescu, Calin Marius Popoiu

**Affiliations:** 1 Department of Pediatric Surgery, ‘Victor Babes’ University of Medicine and Pharmacy Timișoara, 300041 Timisoara, Romania; radueiacob@umft.ro (E.R.I.); mcpopoiu@umft.ro (C.M.P.); 2 Department of Anatomy and Embriology, ‘Victor Babes’ University of Medicine and Pharmacy Timișoara, 300041 Timisoara, Romania; 3 Doctoral School, ‘Victor Babes’ University of Medicine and Pharmacy Timisoara, 300041 Timisoara, Romania; tudor.popoiu@umft.ro; 4 Faculty of Mechanics, Field of Applied Engineering Sciences, Specialization Statistical Methods and Techniques in Health and Clinical Research, ‘Politehnica’ University Timisoara, Mihai Viteazul Boulevard No. 1, 300222 Timisoara, Romania; stoicescu.emil@umft.ro; 5 Discipline of Pathophysiology, “Victor Babeș” University of Medicine and Pharmacy Timișoara, 300041 Timisoara, Romania; bolintineanu.laura@umft.ro

**Keywords:** robot-assisted surgery, minimally invasive surgery, neonatal surgery, surgical devices, pediatric surgery

## Abstract

This narrative review explores the evolution and implications of robotic-assisted surgery in pediatric and neonatal cases, focusing on its advantages, drawbacks, and the specific diseases amenable to this innovative technology. Following PRISMA guidelines, 56 relevant articles from the past five years were selected, emphasizing advancements in precision, reduced trauma, and expedited recovery times for pediatric patients. Despite challenges like cost and training, ongoing research shapes pediatric robotic-assisted surgery, promising improved outcomes. The technology’s benefits include enhanced precision, minimized scarring, and faster recovery, addressing the challenges in delicate pediatric procedures. Challenges encompass cost, training, and instrument design, but ongoing refinements aim to overcome these. This review underscores psychological and musculoskeletal considerations for patients and surgeons. While acknowledging limitations and preferred pathologies, this review outlines the transformative potential of robotic-assisted surgery in reshaping pediatric surgical care. This comprehensive assessment concludes that, despite challenges, ongoing advancements promise a future of enhanced precision and tailored care in pediatric surgery.

## 1. Introduction

Early surgical procedures encountered difficulties due to a restricted comprehension of anatomy, infection management, and anesthetic [1]. In the 20th century, there were advancements in medical procedures, including minimally invasive treatments and, later on, the introduction of robotic-assisted surgery [2]. Presently, surgery has attained unparalleled accuracy and security, owing to state-of-the-art technology such as robotics, improved imaging, and real-time monitoring [1,3].

The presence of surgical conditions for children and neonates is particularly significant because of the delicate nature of pediatric anatomy and the possible long-term consequences on development [4]. When making decisions about pediatric surgery, it is important to take into account not just the immediate clinical results, but also the possible impact on growth, function, and overall wellbeing as children grow into adults [4,5]. Due to the highly specialized nature of pediatric surgery, it is crucial to prioritize clear intervention choices. The enduring ramifications of surgical procedures in youngsters require a meticulous and thoughtful strategy, highlighting the necessity for succinct and knowledgeable decision making to guarantee optimal results for the most juvenile patients [6,7].

In the dynamic landscape of pediatric surgery, traditional open procedures are increasingly complemented by minimally invasive techniques, such as laparoscopy, offering enhanced precision and faster recovery times. Pediatric laparoscopy is crucial for managing surgical conditions in children, offering less invasive procedures with minimal trauma. It results in reduced postoperative pain, shorter hospital stays, and faster recovery. Additionally, it provides improved cosmetic outcomes and enhanced visualization for precise surgical techniques. Its versatility allows for a wide range of procedures across various specialties, benefiting both common and rare pediatric conditions. Overall, pediatric laparoscopy represents a significant advancement in surgical care for children, leading to improved outcomes and quality of life globally [8].

Furthermore, the advent of robotic surgery heralds a new era, where advanced technology augments the surgeon’s skill, enabling unparalleled dexterity and control in delicate procedures. As we embrace these innovations, our commitment remains steadfast: to provide the safest, most effective care for children, ensuring their wellbeing both in the operating room and beyond.

Robotic-assisted surgery combines advanced technology with surgical experience to redefine precision and effectiveness in medical treatments, representing a groundbreaking advancement in modern medical interventions. This procedure utilizes advanced robotic technology and skilled surgeons to provide outstanding precision and control in minimally invasive surgery [9,10]. This innovative method presents a hopeful avenue in various medical fields by integrating sophisticated robots into surgical procedures, leading to improved patient results, shorter recuperation periods, and enhanced surgical capacities [10,11].

The fusion of robotic accuracy and human expertise is the essential element of robotic-assisted surgery. Thanks to the symbiotic interaction between surgeons and robotic arms, surgical procedures may now be conducted with unprecedented precision, facilitated by the employment of highly specialized tools [9,11]. This innovative approach is poised to transform the standards of surgical treatment and expedite the progress that elevates the benchmarks for both the scientific and artistic aspects of medicine as it evolves and discovers novel applications [9,12].

The incorporation of robotic-assisted surgery in pediatric surgery is a significant progress in terms of enhancing precision, minimizing invasiveness, and broadening the range of surgical treatment options for young patients [13,14]. In recent years, there has been a growing trend in the use of robotic systems for pediatric treatments, following their initial development and widespread use in adult surgical settings. This is due to the fact that these technologies offer unique solutions to address the specific challenges associated with operating on smaller, developing anatomies [13,15]. This growing specialty integrates the expertise of pediatric surgeons with modern robotics to revolutionize pediatric surgical procedures, with the aim of improving outcomes and the quality of treatment for children with complex medical conditions [14].

The field of pediatric robotic-assisted surgery holds significant promise, especially for performing precise and intricate procedures on a small scale. The technology’s exceptional precision, flexibility, and little invasiveness make it highly suitable for the rigorous requirements of pediatric surgery, allowing surgeons to manipulate intricate anatomical structures with unparalleled accuracy [16]. This innovative method is revolutionizing pediatric healthcare by reducing surgical trauma, accelerating recovery, and improving long-term outcomes for pediatric patients [13,16].

The objective of this narrative review is to identify the advantages, benefits, and potential drawbacks of robotically assisted surgery for neonates and pediatric patients. Additionally, it aims to determine the specific sorts of diseases that are more suitable for this particular technology.

## 2. Materials and Methods

This research adhered to the PRISMA (Preferred Reporting Items for Systematic Reviews and Meta-Analyses) guidelines for selecting the studies to be included. The present literature review is founded on bibliographic inquiries conducted through the use of MeSH terms (on PubMed) and a combination of human and automated searches in the PubMed database, Google Scholar, and Scopus. A selection was made of the most recent five-year span of publications on pediatric and neonatal robotic-assisted surgery. The most pertinent papers were chosen based on their title, the information presented in their abstract, and a brief review of the full paper. We excluded publications that just provided the abstract, duplicates, articles with no relevant information, and articles published in languages other than English.

A total of two pediatric surgeons with expertise in robotic-assisted surgery carried out the search and selection of the articles in December 2023. Initially, the research papers were hand searched using the following keywords “robotic-assisted surgery” in combination with the following terms: “pediatric population”, “pediatric pathologies”, “neonates”, and “pediatric”, “cost-effectiveness”, “pediatric surgeons skills”. Afterwards, we performed a second search using the MeSH term option that is available in PubMed, with the following terms:

(“Robotic Surgical Procedures”[Mesh]) AND “Pediatrics”[Mesh]

(“Robotic Surgical Procedures”[Mesh]) AND “Infant, Newborn”[Mesh]

((“Robotic Surgical Procedures”[Mesh]) AND “Surgical Procedures, Operative”[Mesh]) AND “Pediatrics”[Mesh]

((“Robotic Surgical Procedures”[Mesh]) AND “Surgical Procedures, Operative”[Mesh]) AND “ Infant, Newborn “[Mesh]

The chosen articles were integrated into a Microsoft Excel table, which included columns for enhanced administration and structure of the review. These columns consisted of the title, authors, year and journal of publication, type of publication, and keywords. Additionally, the table included a summary of the advantages and disadvantages/limitations identified in each study, as well as any other pertinent information that aligned with the objectives of our study.

We chose the most relevant publications based on their advantages and benefits, as well as the researchers’ findings about the method’s limitations. In total, 47 articles were chosen for the literature review since they fulfilled all the requirements. We discussed the main findings and structured the outcomes for clear comprehension, emphasizing the advantages and disadvantages, primary diseases, limitations, costs, and practicability.

**Figure 1:**
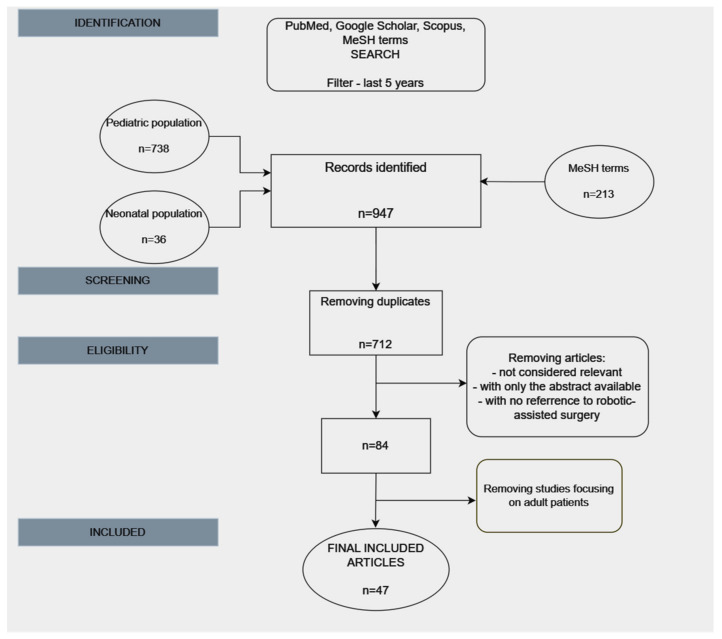
PRISMA diagram explaining the selection process of the included articles.

The process that followed for selecting the articles for the review is summarized in the diagram below (Figure 1).

## 3. The History of Robotic-Assisted Surgery

### 3.1. History of Robotic-Assisted Surgery in Adults

The roots of robotic-assisted surgery can be traced back to the late 20th century, with the development of the da Vinci Surgical System. Introduced in the early 2000s, the da Vinci System marked a significant leap forward in minimally invasive surgery. It comprised robotic arms controlled by a console, offering surgeons enhanced precision and dexterity. The system’s success in urological and gynecological procedures paved the way for its widespread adoption across various surgical disciplines. Over the years, advancements in robotics, coupled with improvements in artificial intelligence and haptic feedback systems, have continually refined robotic-assisted surgery, making it an integral part of modern surgical practices [17,18].

### 3.2. History of Robotic-Assisted Pediatric Surgery

The utilization of robotic-assisted surgery in pediatric patients signifies an important development of this technology into specialized fields. Robotic technology has been a helpful tool in addressing the issues of delicate and precise treatments in pediatric surgery. The initial encounters with robotic systems in pediatric surgery have shown that they are both possible and secure. The reduced-sized robotic tools, along with sophisticated imaging capabilities, enable surgeons to maneuver complex anatomical structures with improved visibility and precision. The lessened invasiveness of robotic-assisted treatments in pediatric patients results in faster recovery periods, reduced postoperative discomfort, and limited scarring, making it a promising direction for the future of pediatric surgical care. Despite the persistent obstacles of cost and training, ongoing research and developments are continuously shaping the field of robotic pediatric-assisted surgery. This progress presents new opportunities for enhanced outcomes in young patients [13].

## 4. Disadvantages/Limitation of Robotic-Assisted Surgery in Neonates and Pediatric Patients

Pediatric robotic-assisted surgery, while offering numerous advantages, is not without its challenges, necessitating a comprehensive consideration of its limitations in clinical practice. A significant concern revolves around the cost implications associated with implementing and maintaining robotic systems in pediatric surgical settings [19]. Studies indicate that the initial investment, ongoing maintenance expenses, and instrument costs can pose financial challenges for healthcare institutions, potentially limiting widespread access and utilization in pediatric surgery [20,21,22].

Training pediatric surgeons to proficiently use robotic systems requires dedicated education and hands-on experience, potentially leading to prolonged surgical durations and increased operative times during the initial learning phase. This aspect underscores the importance of carefully managing the transition to robotic-assisted surgery to optimize patient outcomes [22,23].

The size and design of robotic instruments present additional challenges in pediatric surgeries. Instruments designed for adult procedures might not be optimally tailored for the smaller anatomies of pediatric patients, potentially affecting the precision and adaptability of the system in certain procedures. Maneuvering these instruments within confined spaces or delicate pediatric tissues may pose challenges, emphasizing the need for specialized pediatric adaptations [13,15].

Furthermore, although robotic-assisted surgery is less intrusive, it is not completely devoid of problems in pediatric patients. The possible risks of harm to nearby tissues, nerves, or blood vessels, as well as the possibility of complications arising from the connection of robotic arms, technological glitches, or system faults, although uncommon, necessitate cautious attention during pediatric treatments [24,25]. These documented limitations highlight the importance of ongoing research and refinement to address challenges and ensure the safe and effective application of robotic-assisted surgery in pediatric settings [21,22].

## *5.* Limited Use of Robotic-Assisted Surgery in Specific Pediatric and Neonatal Pathologies

While robotic-assisted surgery has demonstrated efficacy in various pediatric procedures, its adoption remains limited in certain pathologies, primarily due to factors such as instrument size, cost considerations, and the learning curve associated with the technology [26]. Procedures involving very small neonates, where the relative size of robotic instruments may pose challenges, are areas where the adoption of robotic-assisted surgery is limited [27].

Moreover, certain emergency pediatric surgeries, where rapid decision making and interventions are paramount, may not align with the potentially longer setup times associated with robotic procedures. Conditions requiring urgent intervention, such as traumatic injuries or acute abdominal pathologies, may still predominantly rely on traditional surgical approaches [28].

Additionally, orthopedic procedures in pediatric patients, such as limb deformity corrections or spinal surgeries, are areas where the use of robotic-assisted surgery is less common [29]. The unique challenges posed by the dynamic nature of musculoskeletal structures and the need for precise hardware placement may necessitate further advancements in robotic technology tailored specifically for orthopedic applications [30,31].

The following table provides an insightful overview of preferred pathologies for robotic-assisted pediatric surgery, highlighting its efficacy in specific procedures, while also acknowledging its limited use in certain cases due to factors like instrument size, cost considerations, and the learning curve associated with the technology (Table 1).

## 6. Costs, Maintenance, and Feasibility of Robotic-Assisted Surgical Systems

The initial investment encompasses the acquisition of the robotic system itself, with costs varying from hundreds of thousands, to over a million dollars, contingent upon the system’s level of complexity and capabilities. Furthermore, there are costs associated with the installation of the system, adjustments made to the facility to suit the robotic platform, and the acquisition of specialized tools specifically developed for robotic procedures. The financial implications can present difficulties for healthcare facilities, especially those with limited financial matters, raising concerns about the practicality of using robotic-assisted surgery [20,22].

Maintaining a robotic-assisted surgery system involves several aspects to ensure optimal performance and safety. The preventive maintenance of robotic arms, consoles, and associated equipment is crucial to prevent malfunctions and ensure accurate functionality during surgical procedures. Additionally, the training and certification of surgical teams in the use of the robotic system are ongoing requirements for successful and safe utilization. Adequate staffing for system operation, maintenance, and support is vital to address any technical issues promptly and to facilitate the efficient functioning of the robotic-assisted surgery program [22,23,32].

To excel in the dynamic field of robotic-assisted surgery, surgeons and nurses can benefit from specialized courses and workshops designed to enhance their skills and proficiency [33]. Comprehensive training programs often cover various aspects, including system operation, instrument manipulation, and troubleshooting, ensuring healthcare professionals are well equipped to navigate the intricacies of robotic platforms [23,34].

Hands-on workshops play a crucial role, allowing participants to familiarize themselves with the robotic system, practice instrument control, and simulate surgical scenarios [35]. Simulated surgeries on models or virtual platforms offer a risk-free environment for refining techniques and gaining confidence in the use of robotic instruments [23].

Advanced courses delve into specific surgical procedures, offering in-depth insights into the nuances of robotic-assisted interventions. These modules may include gastrointestinal, thoracic, or neurological surgeries, tailoring the training to the diverse needs of pediatric and neonatal patients. Additionally, workshops focusing on adapting robotic techniques to specific anatomical challenges in younger populations contribute to a more holistic skill set [36,37].

Continuous education and refresher courses are essential to keep practitioners updated on the latest advancements and refinements in robotic-assisted surgery. Workshops that incorporate emerging technologies, such as augmented reality or virtual reality simulations, can further enhance the adaptability and expertise of surgeons and nurses in utilizing these innovative tools [20].

## 7. Advantages of Using Robotic-Assisted Surgery in Neonates and Pediatric Patients

One notable benefit of robotic-assisted surgery is the precision and enhanced dexterity afforded by robotic systems, crucial for navigating the intricate anatomical structures of both neonates and children. Studies indicate that the magnified, three-dimensional views and fine-tuned instruments provided by robotic systems enable surgeons to execute more accurate movements in confined spaces, thereby minimizing the risk of damage to delicate tissues [14,38].

The minimally invasive nature of robotic-assisted procedures stands as a significant advantage, particularly in the pediatric and neonatal context. Smaller incisions reduce trauma, blood loss, and postoperative pain, contributing to faster recovery times, compared to traditional open surgeries. This aspect is particularly crucial for both pediatric and neonatal cases, where quicker recoveries play a pivotal role in the overall wellbeing and development of young patients [39].

Furthermore, robotic-assisted surgery offers the advantage of improved visualization and magnification of the surgical field. The high-definition, three-dimensional view provided by robotic systems enables surgeons to operate with greater precision, allowing for meticulous procedures in confined spaces and intricate anatomies, whether in neonates or older children [40].

Quicker postoperative recoveries lead to reduced hospitalization durations, minimizing physical and emotional stress on both pediatric and neonatal patients and their families. Additionally, the application of robotic-assisted surgery often results in improved cosmetic outcomes, with smaller incisions and precise maneuvers contributing to reduced scarring, addressing both the physical and psychological aspects of recovery [13,15].

Studies comparing anesthesia in robotic-assisted surgeries to laparoscopic or open surgeries indicate notable differences in patient management. The minimally invasive nature of robotic surgery contributes to quicker recovery times and less postoperative pain [39]. In contrast, laparoscopic surgeries, while generally requiring less anesthesia than open procedures, may still necessitate more compared to robotic-assisted surgeries due to factors such as pneumoperitoneum maintenance and the need for prolonged operative times. Anesthesia considerations in open surgeries, given the invasiveness and potential for greater tissue disruption, often involve higher doses and more comprehensive pain management strategies [41]. These findings underscore the nuanced approach required in tailoring anesthesia protocols to the specific demands of each surgical modality, emphasizing the importance of individualized patient care [39,42].

Recent studies have shed light on the psychological impact of patients and caregivers in the context of pediatric surgery, comparing robotic-assisted procedures with traditional laparoscopic or open surgeries. The findings suggest that patients undergoing robotic-assisted surgery, particularly children and their caregivers, experience reduced postoperative stress and anxiety. Moreover, the precise movements and enhanced visualization provided by robotic systems may instill confidence in caregivers, alleviating concerns about potential complications [13,43].

In contrast, patients undergoing laparoscopic or open surgeries may exhibit higher levels of postoperative anxiety and discomfort. Larger incisions and the invasive nature of these procedures can contribute to increased pain, potentially impacting the psychological wellbeing of both pediatric patients and their caregivers. The psychological burden associated with the recovery process in traditional surgeries might be higher, emphasizing the potential psychological advantages offered by robotic-assisted techniques in the pediatric population [13,44].

These collective advantages underscore the transformative potential of robotic-assisted surgery in pediatric and neonatal populations, offering safer, more precise, and less invasive solutions for a range of surgical interventions in children of various age groups, including neonates [45].

## 8. Preferred Pathologies for Robotic-Assisted Pediatric Surgery

Numerous studies indicate that robotic-assisted surgery in pediatric settings has shown particular efficacy in certain pathologies, leveraging its precision, enhanced visualization, and minimally invasive nature [46,47]. One notable area where robotic assistance has demonstrated significant advantages is in congenital heart surgeries, especially in intricate procedures involving repairs of congenital cardiac anomalies [48]. The precision offered by robotic systems is particularly beneficial in navigating the complex anatomical structures of the heart in pediatric patients, contributing to improved outcomes and reduced trauma [49,50].

Robotic-assisted surgery has also found utility in cases of pediatric urological pathologies, such as pyeloplasty for ureteropelvic junction obstruction [36,51]. The intricate nature of urological procedures, coupled with the need for precise suturing and reconstruction, makes robotic assistance valuable in achieving optimal results while minimizing invasiveness [52,53,54,55]. Similarly, in pediatric patients requiring gastrointestinal surgeries, such as fundoplication for gastroesophageal reflux disease (GERD), robotic systems offer enhanced dexterity for delicate procedures in confined spaces [56,57,58].

The application of robotic technology has extended to certain pediatric oncological surgeries, particularly in the realm of tumor resections. Studies suggest that the magnified, three-dimensional visualization provided by robotic systems aids in the meticulous removal of tumors, reducing the risk of damage to surrounding healthy tissues. This is especially pertinent in cases where preserving adjacent structures is crucial for the overall wellbeing of the pediatric patient [59].

However, the potential benefits, such as reduced hospital stays, faster recovery times, and improved surgical outcomes, may justify the investment over the long term. Collaborative efforts, training programs, and strategic resource allocation could enhance the feasibility of robotic-assisted surgery in medium-income countries, contributing to advancements in surgical care and patient outcomes [20,60].

A table was created to succinctly outline the benefits and constraints of robotic-assisted pediatric surgery (Table 2).

The table below presents overall comparative and essential information regarding robotic-assisted pediatric surgery in contrast to laparoscopic and open surgery (Table 3).

Currently, pediatric laparoscopy stands out as the optimal approach in the realm of pediatric surgery, offering a blend of minimally invasive techniques and advanced technology that cater specifically to the unique needs of young patients. Compared to traditional open surgery and even laparoscopic surgery, pediatric laparoscopy boasts smaller incisions, high precision, and enhanced visualization, leading to reduced postoperative pain, faster recovery times, and minimal scarring. With its flexibility during operations and broad applicability across various pediatric surgical cases, laparoscopy provides surgeons with the tools to navigate delicate anatomy with precision and perform intricate procedures with ease. While robotic-assisted surgery may offer comparable precision and visualization, its higher initial cost and accessibility limitations make pediatric laparoscopy the more practical choice for pediatric surgical settings, ensuring optimal outcomes and improved quality of life for young patients.

## 9. What Will the Future Bring?

Recent studies highlight significant advancements in robotic-assisted pediatric surgery, ushering in a new era of precision and improved patient outcomes. One notable development is the refinement of robotic systems to accommodate the unique anatomies of pediatric patients. Miniaturization of robotic instruments allows for more precise maneuvers in small spaces, addressing previous challenges associated with the relative size of instruments compared to neonatal and pediatric tissues. These technological enhancements contribute to increased adaptability and efficacy in a broader range of pediatric procedures.

Soon, the evolution of artificial intelligence (AI) is poised to play a pivotal role in enhancing the capabilities of robotic-assisted pediatric surgery. AI algorithms can aid surgeons in real-time decision making, procedural planning, and even automation of certain tasks, reducing the cognitive load on surgeons and potentially improving overall efficiency. The integration of AI-driven technologies holds promise for further refining surgical techniques and expanding the scope of robotic-assisted interventions in pediatrics [47,61].

Additionally, advancements in haptic feedback technology are anticipated to enhance the surgeon’s tactile perception during robotic procedures. Improved haptic feedback can provide a more realistic sense of touch, enabling surgeons to better navigate delicate tissues and perform intricate maneuvers with heightened precision. These developments contribute to the ongoing efforts to bridge the gap between traditional open surgeries and minimally invasive robotic procedures in pediatric patients [47,61].

In the far future, robotic-assisted pediatric surgery is expected to include the integration of telepresence and remote surgery capabilities. This shift in paradigm has the potential to allow proficient pediatric surgeons to do treatments remotely, providing their specialized knowledge to locations with limited access to medical services or during urgent circumstances. The integration of telepresence and robotic technology has the potential to transform the availability of specialized pediatric surgical treatment worldwide, effectively resolving inequalities in healthcare resources.

Moreover, the fusion of nanotechnology and robotics has the capacity to provide revolutionary advancements in the field of pediatric surgery. The potential exists to create nanoscale robotic devices capable of maneuvering through complex anatomical systems with unparalleled accuracy, thereby revolutionizing targeted drug administration, tissue restoration, and diagnostic procedures. These futuristic advancements highlight the transformative path of robotic-assisted pediatric surgery, offering improved accuracy, increased capabilities, and better availability for young patients globally.

A table with the main information of the studies included in this review can be found below (Table 4).

## 10. Discussions

### 10.1. Clinical Scenarios

Robotic-assisted surgery has demonstrated notable advantages in specific clinical scenarios within the pediatric population, offering innovative solutions for complex procedures. In pediatric urology, surgeries like pyeloplasty or ureteral reimplantation require delicate maneuvers in small spaces. Robotic platforms provide enhanced visualization and dexterity, enabling surgeons to perform intricate tasks with greater precision, reducing the risk of complications and improving outcomes. The minimally invasive nature of robotic-assisted procedures is particularly beneficial for pediatric patients, promoting a faster recovery and minimizing postoperative discomfort [36].

The complexity of cardiac design presents special issues in congenital heart operations performed on pediatric patients. Robotic-assisted techniques offer benefits in specific cardiac procedures, enabling surgeons to maneuver through the complexities of the heart with enhanced accuracy. This is especially pertinent in situations such as atrial septal defect closures, where the capacity to operate in limited places is vital. The possibility of using smaller incisions and minimizing stress plays a significant role in promoting speedier healing, which is crucial for the susceptible juvenile population [49].

In pediatric colorectal surgeries, such as pull-through procedures for anorectal malformations, robotic-assisted surgery offers advantages over traditional approaches. The flexibility and articulation of robotic instruments facilitate complex dissections, and the three-dimensional visualization enhances the surgeon’s spatial awareness, aiding in meticulous anastomoses. This can lead to reduced postoperative complications and shorter hospital stays for pediatric patients undergoing colorectal interventions [56].

While robotic-assisted surgery holds promise in specific pediatric clinical scenarios, its application requires careful consideration. Factors such as patient age, size, and the complexity of the procedure influence the decision to opt for a robotic approach. Continued research and advancements in pediatric robotic surgery aim to expand its applications, offering tailored solutions for the unique challenges posed by surgical interventions in the pediatric population.

### 10.2. Advantages, Limitations, and Considerations

Robotic-assisted surgery in pediatric and neonatal populations presents both notable advantages and inherent challenges. On the positive side, the precision and enhanced dexterity offered by robotic systems are crucial for navigating intricate anatomical structures in young patients. The minimally invasive nature of these procedures, characterized by smaller incisions, contributes to reduced trauma, blood loss, and faster recovery times, which is particularly significant in pediatric cases where swift recuperation is paramount for wellbeing and development. Improved cosmetic outcomes, owing to smaller incisions and precise maneuvers, address both physical and psychological aspects of recovery in these vulnerable populations.

The decision between robotic-assisted pediatric surgery and traditional laparoscopy or open surgery carries substantial psychosocial consequences for both patients and caregivers. Research indicates that patients, particularly youngsters, who have robotic treatments experience a decrease in postoperative tension and anxiety. The reduced invasiveness, smaller incisions, and decreased postoperative pain contribute to a more favorable psychological experience. The utilization of robotic devices improves accuracy and provides caregivers with clear visual representation, boosting their confidence and reducing worries about potential consequences. However, patients who undergo laparoscopic or open operations may encounter elevated levels of postoperative anxiety and discomfort as a result of larger incisions and the intrusive nature of the procedures. It is essential to acknowledge these psychological factors in order to provide patient-centered treatment, highlighting the importance of additional studies to educate healthcare workers and enhance the overall experience for pediatric patients and their families.

However, the adoption of robotic-assisted surgery in pediatric settings is not without its limitations. The substantial initial investment, maintenance expenses, and high instrument costs associated with robotic platforms can pose financial challenges for healthcare institutions, limiting accessibility. The learning curve involved in training pediatric surgeons for the proficient use of robotic systems may result in prolonged operative times, potentially affecting patient outcomes. Additionally, the size and design of robotic instruments, initially designed for adults, can be suboptimal for the smaller anatomies of pediatric patients, impacting the precision and adaptability of the system in certain procedures.

Lastly, it is crucial to comprehend and address the musculoskeletal difficulties that surgeons have after undergoing robotic-assisted surgery in order to ensure the long-term viability and welfare of these healthcare practitioners. Recent studies have shown an increasing interest in the consequences of musculoskeletal illnesses in surgeons who conduct robotic-assisted surgery, laparoscopic procedures, and open surgery. Research indicates that surgeons who perform lengthy robotic-assisted surgeries may have musculoskeletal discomfort, including neck and back pain, as well as ergonomic difficulties associated with the console arrangement [62,63]. The immobile and occasionally restricted positioning used during robotic surgery may lead to these problems. To address these issues, effective measures include providing comprehensive instruction in ergonomics, scheduling regular breaks, and optimizing the console arrangement to minimize physical strain. Similarly, laparoscopic surgery, although typically seen as less physically strenuous than open surgery, presents its own set of difficulties. Prolonged maintaining of a stooped position and the use of long-handled devices during laparoscopy can result in musculoskeletal strain, namely in the upper back and neck. Research highlights the significance of providing laparoscopic surgeons with comprehensive ergonomic training and promoting their awareness to mitigate the potential hazards of musculoskeletal problems. By incorporating ergonomic concepts, such as the use of adjustable operating tables and well-designed instruments, it is possible to create a working environment that is both comfortable and sustainable. On the other hand, surgeons who do open surgery may encounter specific musculoskeletal difficulties. The physically strenuous characteristics of open operations, which frequently entail lengthy incisions and manual tissue manipulation, can lead to diseases such as back pain and joint strain. Methods for dealing with these obstacles encompass the utilization of ergonomic operating tables, ensuring suitable positioning of the surgeon, and implementing regular training regimens to augment strength and flexibility [62,63].

In the near future, several improvements could enhance the feasibility and accessibility of robotic-assisted surgery in pediatric and neonatal settings. Firstly, advancements in robotic technology tailored specifically for smaller anatomies could address the current limitations posed by instrument size. Developing smaller robotic instruments designed specifically for neonatal and pediatric patients would improve maneuverability within confined spaces and delicate tissues, allowing for more precise and adaptable surgical interventions. Additionally, innovations in instrument design that offer greater flexibility and range of motion could further enhance the capabilities of robotic systems, facilitating complex procedures with improved outcomes.

Moreover, efforts to reduce the costs associated with robotic-assisted surgery systems could broaden access to this innovative technology. Continued research and development focused on streamlining manufacturing processes and optimizing resource utilization could lead to more affordable robotic platforms. Additionally, exploring alternative financing models, such as leasing or shared access programs, could mitigate the financial burden on healthcare facilities and make robotic-assisted surgery more financially feasible. Collaborative initiatives between industry stakeholders, healthcare institutions, and regulatory bodies to establish cost-effective pathways for acquiring and maintaining robotic systems would be instrumental in expanding access to this transformative surgical approach for pediatric and neonatal patients.

## 11. Limitations of the Study

This narrative review on pediatric and neonatal robotic-assisted surgery, while invaluable, might encounter several limitations that we consider important to share with other researchers.

Addressing the landscape of pediatric and neonatal robotic-assisted surgery poses several challenges stemming from the limited comparative data available. The scarcity of direct comparative studies across surgical modalities in these populations hinders the depth of insights into the relative effectiveness of robotic-assisted surgery versus traditional or laparoscopic approaches. Heterogeneity in methodologies, sample sizes, and specific pathologies within the literature further complicates synthesizing findings, introducing variability that may impact the overall reliability of conclusions drawn from the review. Additionally, susceptibility to publication bias may skew the representation of evidence, potentially favoring studies with positive or statistically significant results, thus influencing the perceived effectiveness of robotic-assisted procedures.

Temporal constraints pose another challenge, as the dynamic field of pediatric and neonatal robotic-assisted surgery evolves rapidly, potentially outpacing the available literature. Reviews may struggle to capture the most recent developments, limiting their ability to provide a comprehensive overview of the latest advancements. Furthermore, the limited availability of long-term follow-up data, attributable to the recent adoption of robotic-assisted surgery in these contexts, poses a challenge in evaluating the durability of outcomes and potential long-term complications associated with these procedures. Assessing the overall quality of evidence across studies with varying levels of methodological rigor and reporting standards adds another layer of complexity to this review, impacting its reliability and the strength of the conclusions drawn regarding the efficacy of robotic-assisted surgery in pediatric and neonatal populations.

## 12. Conclusions

Pediatric and neonatal robotic-assisted surgery represent a pivotal advancement in the landscape of pediatric and neonatal surgical care. The amalgamation of enhanced precision, reduced invasiveness, and potential for quicker recoveries underscores its transformative potential in reshaping the future of medicine for young patients. While facing limitations such as cost, instrument size constraints, and the learning curve for surgeons, the advantages in terms of improved surgical accuracy, minimized trauma, and shorter recovery times highlight the promising trajectory of this technology.

While pediatric and neonatal robotic-assisted surgery represents a significant advancement with transformative potential in reshaping surgical care for young patients, it currently faces limitations such as cost, instrument size constraints, and the learning curve for surgeons. Despite these challenges, the technology holds promise for improving surgical accuracy, minimizing trauma, and shortening recovery times. However, for the moment, laparoscopy remains the method of choice in many pediatric surgical settings due to its established efficacy, versatility, and broader applicability across various procedures. As technology continues to evolve and research progresses, the future may see robotic-assisted surgery becoming more widely adopted, offering enhanced outcomes and further revolutionizing pediatric surgical care.

## 13. Future Directions of Studies

Future research directions in pediatric and neonatal robotic-assisted surgery encompass a broad spectrum of areas. One key focus is the expansion of surgical applications, emphasizing the feasibility and outcomes of employing robotic systems in diverse procedures such as gastrointestinal, thoracic, and neurological interventions. This necessitates the exploration of new techniques and adaptations tailored to the specific anatomies of pediatric and neonatal patients, ultimately enhancing the versatility and applicability of robotic surgery within these populations.

Simultaneously, a crucial aspect involves the refinement of robotic systems to better align with the unique requirements of pediatric and neonatal surgery. Research efforts can concentrate on the development of smaller, more adaptable instruments specifically designed for the delicate anatomies of young patients. Improvements in the ergonomics, visualization, and maneuverability of robotic platforms, particularly in the confined-spaces characteristic of pediatric surgeries, are vital for optimizing surgical outcomes and minimizing potential complications.

Another crucial avenue for future research lies in comprehensive comparative studies and long-term outcome assessments. Investigating recurrence rates, functional outcomes, and patient satisfaction across various surgical modalities in pediatric and neonatal care can provide valuable insights into the overall effectiveness and durability of robotic-assisted surgery compared to the traditional approaches. Additionally, a focus on enhancing training programs for pediatric surgeons in robotic-assisted techniques, including standardized protocols and simulation-based learning tools, ensures the proficiency of surgeons in utilizing these technologies safely. Evaluating the learning curve and skill retention among pediatric and neonatal surgeons is essential for the widespread and proficient adoption of robotic-assisted surgery. Lastly, addressing the cost-effectiveness and economic impact of implementing robotic systems in pediatric and neonatal care settings is crucial for optimizing resource utilization and ensuring equitable access to this innovative technology across diverse healthcare environments.

Finally, prospective future study could focus on conducting a comprehensive comparison between two or more distinct, robotic-assisted surgery systems or technologies, aiming to provide valuable insights into their relative efficacy, safety, and performance across various surgical procedures. Such a study could delve into parameters such as precision, maneuverability, ease of use, and adaptability to different surgical scenarios. Analyzing the strengths and limitations of each system, along with their respective learning curves for surgeons, would contribute to a more nuanced understanding of the practical implications of these technologies in clinical settings. The outcomes of such a study would not only inform surgeons, healthcare practitioners, and healthcare administrators about the comparative advantages and disadvantages of different robotic systems but also guide future advancements in the field. This research could influence the direction of robotic-assisted surgery development, fostering innovation and refinement to address identified shortcomings and optimize the overall effectiveness of these technologies in improving patient outcomes.

## Figures and Tables

**Table 1:** Robotic-assisted pediatric surgery preferences and limitations in the pathology spectrum.

Pediatric Pathologies	Preference for Robotic- Assisted Surgery	Limited Use of Robotic- Assisted Surgery
Congenital heart surgeries	Repair of congenital cardiac anomalies	-
Urological surgeries	Pyeloplasty for ureteropelvic junction obstruction	Neonatal procedures with very small patients
Gastrointestinal surgeries	Fundoplication for gastroesophageal reflux disease (GERD)	Emergency surgeries requiring rapid interventions
Oncological surgeries	Tumor resections for certain pediatric oncological cases	Orthopedic procedures (limb deformity corrections, spinal surgeries)

**Table 2:** Advantages and disadvantages of robotic-assisted pediatric surgery.

Advantages of Robotic-Assisted Surgery	Disadvantages of Robotic-Assisted Surgery
Enhanced precision and dexterity	High initial setup and maintenance costs
Minimally invasive procedures	Steeper learning curve for surgical teams
Reduced postoperative pain	Limited tactile feedback
Smaller incisions and scars	Size and design constraints of instruments
Faster recovery times	Potential for technical malfunctions
Improved visualization	Increased operative times initially
Lesser blood loss	Limited availability in some settings
Reduced risk of complications	Lack of standardized training protocols
Shorter hospital stays	
Improved cosmetic outcomes	

**Table 3:** Comparative data between robotic-assisted surgery and laparoscopic and open surgery.

Evaluated Criteria	Robotic-Assisted Surgery	Laparoscopic Surgery	Open Surgery
Incision size	Smaller incision	Small incision	Large incision
Precision/visualization	High precision and visualization	Good precision and visualization	Lower precision and visualization
Flexibility during operation	High flexibility	Limited flexibility	Standard flexibility
Learning curve	Moderate learning curve	Steeper learning curve	Standard learning curve
Postoperative pain	Reduced postoperative pain	Moderate pain	Higher pain
Recovery time	Faster recovery	Fast recovery	Slower recovery
Postsurgical scars	Minimal scarring	Small scars	Large scars
Risk of complications	Lower risk	Moderate risk	Higher risk
Cases applicability	Broad applicability	Applicable in many cases	Standard cases
Costs, accessibility	Higher initial cost, accessibility in developed settings	Moderate cost, accessible	Lower cost, accessible

**Table 4:** The main information of the included studies.

Aspect	Advantages	Disadvantages/Limitations	Preferred Pathologies	Limited Use in Specific Pediatric and Neonatal Pathologies
Precision and dexterity	Enhanced precision and dexterity for intricate anatomical structures	Initial investment, maintenance costs, and instrument expenses can be challenging for healthcare institutions	Congenital heart surgeries	Neonates with very small sizes where relative instrument size poses challenges
Minimally invasive procedures	Smaller incisions reduce trauma, blood loss, and postoperative pain	Learning curve for surgeons may lead to prolonged surgical durations	Pediatric urological pathologies (e.g., pyeloplasty)	Emergency pediatric surgeries requiring rapid interventions, like traumatic injuries or acute abdominal pathologies
Improved visualization	High-definition, three-dimensional view for better visualization	Size and design of robotic instruments may not be optimally tailored for pediatric anatomies	Gastrointestinal surgeries (e.g., fundoplication)	Orthopedic procedures in pediatric patients (e.g., limb deformity corrections or spinal surgeries)
Shorter hospital stays	Quicker recovery times contribute to reduced hospitalization durations	Possible risks of harm to nearby tissues or complications from technological glitches	Pediatric oncological surgeries (e.g., tumor resections)	
Anesthesia considerations	Less anesthesia required due to minimally invasive nature	Risks of harm to nearby tissues, nerves, or blood vessels must be cautiously managed		
Psychological impact	Reduced postoperative stress and anxiety for patients and caregivers	Learning curve for surgeons and potential complications from system faults		
Feasibility in medium-income countries	Potential benefits may justify long-term investment despite upfront costs	Financial and logistical challenges may limit accessibility		
Future developments	Miniaturization of robotic instruments for better adaptability in pediatric procedures	Integration of AI for real-time decision making, procedural planning, and automation of tasks	Refinement of robotic systems to accommodate pediatric anatomies	Integration of telepresence and remote surgery capabilities, potential fusion of nanotechnology the far future

## Data Availability

Not applicable.
